# Editorial: Immune Responses of the Mucosal Epithelium in Chronic Lung Diseases

**DOI:** 10.3389/fimmu.2020.626437

**Published:** 2020-12-18

**Authors:** Christian Herr, Marc Chanson, Loïc Guillot

**Affiliations:** ^1^Department of Internal Medicine V-Pulmonology, Allergology and Critical Care Medicine, Saarland University, Homburg, Germany; ^2^Department of Cell Physiology & Metabolism, Faculty of Medicine, University of Geneva, Geneva, Switzerland; ^3^Sorbonne Université, INSERM UMR S 938, Centre de Recherche Saint-Antoine (CRSA), Paris, France

**Keywords:** lung, epithelium, chronic lung disease, innate immunity, host defense

The airway epithelium plays a prominent role in protecting us against detrimental agents since it is continually exposed to particles that are potentially harmful for the lungs including microorganisms, dust and air pollutants. However, *via* mucociliary clearance and coughing, these agents are usually eliminated and airway integrity is protected from these potentially damaging attacks. Until recently, the epithelium of the respiratory tract was only seen as a physical barrier, macrophages being considered as the main immune sensor of aggression. Although its structural integrity is essential, it is now well established that the airway epithelium plays a major role in triggering an innate immune response to protect the lung from infection and injury in various chronic respiratory diseases. A defect of these protections in the airways can induce some diseases as seen with the current health crisis caused by Severe Acute Respiratory Syndrome (SARS)-CoV2. Indeed, airway epithelium is playing a crucial role in the host defense against this virus (1). Defects in these mechanisms are also associated with chronic lung pathologies including cystic fibrosis (CF), chronic obstructive pulmonary disease (COPD) and asthma. The 14 articles of this Research Topic highlight the latest advances regarding the role of the airway epithelium immune response in chronic respiratory disease.

In a mini-review of the literature, Guo-Parke et al. expose the cellular and molecular mechanism involved in COPD. COPD is a complex disease and it is estimated that around 50% of COPD acute exacerbations are related to respiratory viral infection. In this context, the authors especially address how respiratory viruses altered the immune response of the airway epithelium in the pathogenesis of exacerbations. They summarize the contribution of T cell exhaustion, NF-κB, TLR, EFGR, IFNs, and inflammasome.

Cystic fibrosis patients are often infected or colonized by the bacteria *P. aeruginosa*. It has been shown that *P. aeruginosa* acquire mutations during colonization, which promote their resistance, often associated with the acquisition of antibiotic resistance. Antimicrobial peptides (AMPs) are endogenous antibiotically active factors, produced by many different types of cells. Their antimicrobial activity mostly depends on their interaction with bacteria or host cell membranes but is also influenced by pH and ionic strength. Geitani et al. review the latest findings on the potential of antimicrobial peptides as therapeutics in CF.

Although bacterial pathogens play a prominent role in many pulmonary diseases, virus sometimes outperform their pathogenicity or predispose the host to a more severe course of the disease. This is the case for CF patients, who are often infected with viruses including *Influenza A* virus. In an original study Villeret et al. address what is the result of a concomitant infection with IAV and *P. aeruginosa*. They demonstrate with epithelial cells *in vitro* and *in vivo* that IAV promotes the deleterious effects of a secondary infection with *P. aeruginosa*. This damaging response is characterized by an increase of MMP9 activity and its inhibition supports lung resilience with no effect on the bacterial clearance. Also, the authors showed that IAV subverts the host response by inhibiting the antimicrobial/antiprotease molecule elafin/Trapin 2 transcript, known to possess beneficial anti-inflammatory properties. Altogether, the results of this study suggest that restoring tissue resilience could be a successful strategy in a coinfection situation. Montgomery et al. investigate rhinovirus (RV) induced airway epithelial cell necrosis in young children with CF. RV is indeed the commonest respiratory virus detected in the CF airways. These authors provide data that RV infection in airway epithelial cells from children with CF leads to more necrotic cell death and a higher IL-1R signaling. IL-1R in turn is a known driver for airway neutrophilia and mucin production. Thus, using IL1-Ra could mitigate the severity of the disease. Infection usually results in a complex signature of differentially expressed genes, not only involving IL-1 related pathways. With the advent of multiplex analysis tools, the complex nature of bacterial and viral infections has become even more evident. Applying a transcriptomic approach on primary tracheal CF human airway epithelial cell cultures infected with RV, Ling et al. provide detailed analysis of the biological pathways that are differently induced in comparison to non-CF cells. Their work confirmed the findings from Montgomery et al. showing that in response to RV infection, the genes for IL-1 signaling and mucin glycosylation were mostly dysregulated in fully differentiated CF airway epithelial cells.

Another hallmark of CF is the exaggerated neutrophil-dominated innate immune response. Indeed, Cystic fibrosis transmembrane receptor (CFTR) is broadly expressed on epithelial cells and cells of myeloid origin. Neutrophils contribute to the deleterious evolution of the CF lung disease. Cabrini et al. extensively review the hypotheses that there is an abnormal airway epithelial cell response in CF. This is due to the receptor-activated intracellular signaling pathways and the influence of epigenetic regulation of key chemokines. Laucirica et al. contributed a comprehensive overview on the latest progress of model systems to investigate mucosal inflammation in CF using cell and animal models. The focus of their review is on the function of neutrophils and their aberrant activation in the course of CF. Molloy et al. extends the topic to the secretome of *Stenotrophomonas maltophilia*, a Gram-negative opportunistic pathogen that can chronically colonize the lungs of people with CF and is associated with lethal pulmonary hemorrhage in immunocompromised patients. They show how bacterial proteases impair the airway epithelium integrity by disrupting the tight junctional complex in CFBE41o- bronchial epithelial cells, particularly ZO-1 and occludin.

In addition to viruses and bacteria, the airway epithelium is exposed to fungi including *Aspergillus fumigatus*. This filamentous fungus is found in the environment and can be pathogenic in immunocompromised patients or patients with altered mucociliary clearance such as CF patients. Bigot et al. reviewed the contribution of airway epithelial cells in the host immune response against *A. fumigatus*. The models to study *A. fumigatus*-airway epithelium are depicted as well as the different step of the host immune response: recognition, internalization, and host response of the bronchial epithelium.

Asthma is a complex respiratory disease with variable and complex symptoms. Frey et al. made a very comprehensive review describing the detrimental role of the airway epithelium in the formation, progression and acute exacerbation of asthma. They especially describe how the barriers (mucus and periciliary layers) and immune function (i.e. sIgA) of the airway epithelium are altered is asthma pathogenesis. In asthma, epithelial mesenchymal transition (EMT), which is necessary during lung development, is detrimental during asthma pathogenesis. Sun et al. provide data, that the exposure to house dust mite leads to an increased expression of IL-33 and CD146, which in turn induce EMT. The regulation of gene expression comprises multiple layers. Transcription factors, epigenetic modifications and small RNA-species potentially influence each other and in consequence the expression of their target genes. Epigenetically regulated gene expression in the context of asthma (or any other pathologic condition) is very interesting, since it is potentially hereditary and may impact the health of future generations. Besides epigenetic modifications, small RNA-species modify gene transcription. Alhamwe et al. review the latest findings that proof the influence of epigenetic modifications on the pathogenesis of asthma associated pulmonary inflammation.

Particles and compounds derived from cigarette smoke (CS), traffic, industry or open hearth play an important role in the development of many so-called “disease of civilization” like asthma, allergy, and COPD. Singh et al. contribute a very interesting study, highlighting physiological mechanisms by which maternal exposure to CS downregulate H_2_S synthesizing enzymes in preclinical models and human placentas. H_2_S synthesizing enzymes play a role in EMT during lung development and asthma pathogenesis. Interestingly, the CS-induced inhibition of H_2_S synthesizing enzymes was transmitted to the F2 progeny and may increase risk for the development of asthma or bronchopulmonary dysplasia.

Interplay between metabolism and immunity/inflammation is an emerging domain of research. In this context, several associations between vitamin D deficiency and respiratory diseases and infections have been described. Schrumpf et al. reviewed the current knowledge about mucosal vitamin D metabolism and its signaling in chronic lung disease. Particularly, its metabolism in health and chronic inflammatory diseases is described, followed by the description of its protective effects on mucosal homeostasis. Finally, the last strategies of treatment with vitamin D are described.

## Author Contributions

CH, MC, and LG wrote the editorial. All authors contributed to the article and approved the submitted version.

## Conflict of Interest

The authors declare that the research was conducted in the absence of any commercial or financial relationships that could be construed as a potential conflict of interest.
